# Case Report: Anesthetic management for Cesarean section in a parturient with unspecified inherited bleeding disorder

**DOI:** 10.12688/f1000research.16097.2

**Published:** 2018-11-29

**Authors:** Li Li, Jill M. Johnsen, Chau X. Doan, Laurent A. Bollag

**Affiliations:** 1 Department of Anesthesiology & Pain Medicine, University of Washington, Seattle, WA, 98195, USA; 2 Department of Medicine - Hematology, University of Washington, Seattle, WA, 98195, USA

**Keywords:** Anesthesia, Spinal, Cesarean section, Parturient, Bleeding disorder

## Abstract

Neuraxial anesthesia, as the standard of care for Cesarean deliveries, is associated with decreased blood loss. However, parturients with inherited bleeding disorders are at increased risk for epidural hematomas. A small retrospective study has shown that parturients with known factor deficiencies can safely undergo neuraxial anesthesia once the specific factors are replenished. We present a patient who had a considerably increased risk of peripartum bleeding from an unspecified inherited bleeding disorder and was provided a successful neuraxial anesthetic without complications. We discuss the multidisciplinary approach among the surgeons, anesthesiologists, hematologist, and nursing staff to maximize patient safety and comfort.

## Introduction

Neuraxial anesthesia, which encompasses both spinal and epidural anesthesia, has been established as a safe and effective regional anesthetic technique for the obstetric patient. Although considered the standard of care for Cesarean deliveries (CDs), this technique carries a small but potentially devastating risk of neuraxial bleeding complications (e.g. spinal epidural hematomas (SEH)) that may result in significant neurological injury or compromise. The risk of SEH in obstetric patients from neuraxial anesthesia is estimated to be 1:168,000 ^1^. Parturients with coagulopathy or bleeding diathesis are at increased risk of neuraxial hematomas ^2, 3^. Although a recent study has provided a management outline for patients with unclassified bleeding disorders undergoing procedures ^4^, no specific recommendations were made for CDs, which is unique in that hematologic management directly impacts anesthetic management. In fact, although parturients with known hemostatic deficiencies can often safely undergo neuraxial anesthesia once the hemostatic defects are corrected ^5– 7^, due to the lack of recommendations, neuraxial anesthesia is often avoided in these patients, as in patients with other coagulopathies ^8^. Physiological hypercoagulability and larger lumbar epidural space during pregnancy may explain why SEH in parturients are rare. While older guidelines are more conservative regarding platelet count cutoffs for neuraxial anesthesia, the SEH risk in patients with platelet counts less than 70,000 remains unknown ^9^.

Here, we present the anesthetic management and safe utilization of neuraxial anesthesia for a CD in a patient with an unspecified inherited bleeding disorder.

## Case description

A 39-year-old G6P3 with an unspecified inherited bleeding disorder presented at 37-6/7 weeks gestational age for a fourth repeat scheduled CD in the setting of fetal macrosomia. The patient had a diagnosis of an unspecified bleeding disorder. Prior to diagnosis, her bleeding history consisted of easy bruising, gingival bleeding, heavy menstrual bleeding, and multiple post-operative hemorrhagic complications after previous surgeries. These complications included hemorrhagic compartment syndrome after an ankle surgery and bleeding complications with all three prior deliveries including persistent vaginal bleeding after her first two CDs and intra-abdominal hemorrhage after her third CD. Although she reported having had epidurals during her prior pregnancies, these anesthetic records were unavailable. Her family history is positive for mucocutaneous bleeding including maternal heavy menstrual bleeding and death of her maternal grandmother secondary to postpartum hemorrhage. Prior to this pregnancy, she was referred for evaluation of a bleeding disorder in advance of cervical spine surgery. Her extensive coagulation laboratory evaluation was unremarkable, including normal complete blood count and smear, activated partial thromboplastin time, prothrombin time, thrombin time, von Willebrand factor (VWF) parameters, fibrinogen activity, platelet aggregation studies (including ristocetin-induced platelet aggregation), platelet function assay, factor XIII level, and rotational thromboelastography. There was good documentation of her prior bleeding and no suspicion for other disorders, such as endocrine or connective tissue disorders. She was diagnosed with an unspecified inherited bleeding disorder. The differential diagnosis for her hemostatic defect included rare congenital bleeding disorders such as undetected VWF qualitative dysfunction or undetected defects in fibrin, fibrinolysis, or platelet function. She received prophylactic fresh frozen plasma (FFP), cryoprecipitate, platelets, and anti-fibrinolytic treatment as prophylaxis for her cervical spine surgery and achieved good hemostasis without complication.

Upon presentation for delivery, her laboratory values were unremarkable: hematocrit (Hct) 30%, platelets 169 × 10 ^3^/ml, international normalized ratio (INR) 1.1, partial thromboplastin time (PTT) 30 s, fibrinogen activity 461 mg/dl, and a thromboelastogram (TEG) within normal parameters. Prior to her delivery, a multi-disciplinary care plan, including hematology, anesthesiology, and obstetric services was established and a delivery plan that balanced patient safety and birthing preferences agreed upon; contingency plans for transfusion and anatomic control of bleeding were in place in the event of obstetric hemorrhage. The risks and benefits of regional anesthesia in the setting of a unremarkable airway exam (Mallampati Score 2, normal head flexion and extension, and normal mouth opening), were discussed at length with the patient, acknowledging the diagnostic challenges and lack of a useful laboratory monitoring test.

**Figure 1.**
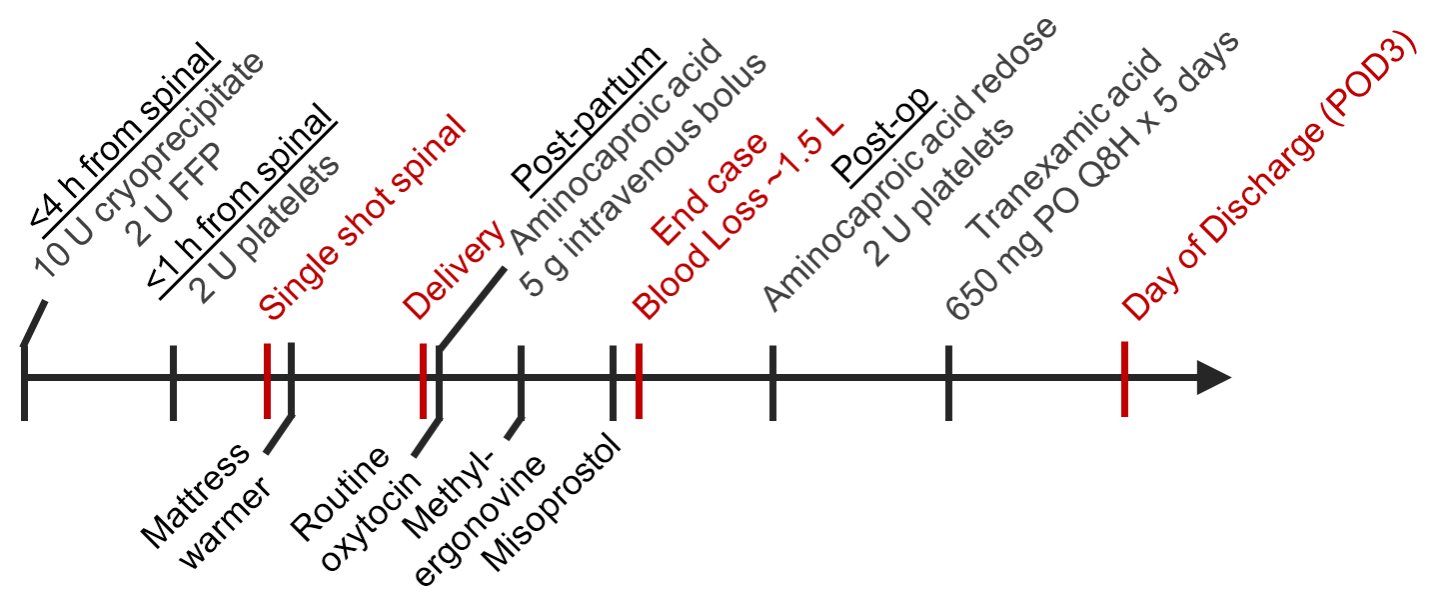
Case management timeline. Hematologic interventions for the planned Cesarean section are shown on top, whereas the intraoperative interventions to reduce bleeding risks are shown on the bottom of the arrow. Case events are shown in red.

The patient prophylactically received 2 units of FFP, 10 units of cryoprecipitate within 4 hours prior to CD, and 2 units of platelets immediately before the placement of her routine single shot spinal anesthetic (consisting of 12.5 mg hyperbaric bupivacaine, 10 mcg fentanyl, and 100 mcg preservative-free morphine). Following the delivery of a healthy infant, intravenous aminocaproic acid was immediately started and routine oxytocin was infused (at an approximate rate of 15 IU per hour). Poor uterine tone was noted after delivery, which improved after single dose of 200 ug IM methylergonovine and 800 mg buccal misoprostol administration. The estimated blood loss was 1.5 liters. In response to her higher-than-average blood loss, 2 additional units of platelets were given in the immediate postoperative period. Deep venous thrombosis prophylaxis consisted of sequential compression devices until ambulation; low molecular weight heparin was avoided. The patient was closely monitored for neuraxial hematoma. Her postoperative labs were notable for Hct 22%, platelets 175 × 10 ^3^/ml, INR 1.1, PTT 28 s, and fibrinogen activity 462 mg/dl. Additionally, TEG results during and after the CD remained normal. The patient continued intravenous aminocaproic acid therapy postpartum, and transitioned to oral tranexamic acid for 5 days. She had an uneventful recovery and was discharged 3 days after surgery. The case management timeline and dosing regimen are shown in Figure 1.

## Discussion

This patient’s unspecified inherited bleeding disorder placed her at considerably increased risk of peripartum bleeding with risks compounded by fetal macrosomia, multiple prior CDs, and a personal history of obstetric hemorrhage in the absence of prophylaxis. Typically, for a well-defined bleeding disorder prophylaxis can be used with appropriate repletion of the respective pathway components. For patients with unspecified bleeding disorders, it is not possible to know if the hemostatic defect has been adequately corrected by the prophylaxis treatment options available. However, in this case her spine surgery history could inform an effective prophylaxis regimen. For both procedures, the goal was to seek balanced hemostatic replacement for a rare hemostatic defect that was clinically established but undetectable by laboratory testing. For her spine surgery cryoprecipitate was administered with the goal of modest replacement treatment of several exogenous coagulation factors (fibrinogen, FXIII, VWF), which were in her bleeding disorder differential diagnosis. The addition of VWF replacement or ddAVP/desmopressin was thought to confer more risk than benefit because no laboratory defect in VWF (qualitative or quantitative) was found to support a diagnosis of von Willebrand disease (VWD). Although there are rare qualitative VWF defects known to cause VWD that we cannot test for clinically ^10^, there remained other possible hemostatic defects in her differential diagnosis that VWF replacement would not correct. Conversely, there is a concern that a VWF infusion would result in unacceptably high levels of functional VWF, increasing her risk for thrombosis, as VWF is already markedly elevated by normal pregnancy ^11, 12^. 

We considered using ddAVP, not to correct a VWF defect, but to improve hemostasis in qualitative platelet disorders (which are in her differential diagnosis). However, ddAVP is rarely used unless there is an established laboratory response correcting a hemostatic defect, which is not the case here as her laboratory workup was normal. Specifically, for her spine surgery, ddAVP seemed suboptimal due to its short duration of action compared to the duration of hemostatic control needed after surgery. Similarly, for her delivery, ddAVP was not used because we already had a known, effective hemostatic regimen. Furthermore, even in established cases of type 1 VWD, ddAVP is used sparingly during delivery due to tachyphylaxis (ddAVP has a short effective treatment duration relative to the treatment duration needed to prevent postpartum hemorrhage) and increased risk for hyopnatremia.

Good communication and detailed hematology recommendations regarding the type, dose, and timing of blood product administration were critical in the planning and delivery of a safe spinal anesthetic for this patient.

Despite the potential for prolonged duration of surgery in the setting of a fourth CD, a spinal anesthetic was preferred over a combined spinal epidural to minimize the risk of an epidural catheter-associated hematoma ^13, 14^. Although spinal clonidine can extend anesthestic duration, it was not used because it can cause prolonged hypotension, potentially complicating patient management. General anesthesia, even with comparable safety in obstetrics ^15^, was reserved for a failed spinal anesthesia or bleeding emergency to optimize maternal uterine tone ^16^ and fetal neurological adaptation, both of which are affected by the use of volatile anesthetics, and to allow for early maternal-infant skin-to-skin bonding, honoring the mothers wish. Also important, the patient desired a regional anesthetic.

Other management considerations to guide appropriate planning and reduce the risk of significant perioperative bleeding include: early pre-anesthetic consultation, pre-operative hematology consultation, expeditious use of intraoperative uterotonics, and maintenance of normothermia to prevent hypothermic coagulopathy. Co-loading with crystalloid or colloid prior to initiation of neuraxial anesthesia can attenuate hypotension—commonly observed with spinal-related sympathectomy—which may be exacerbated in the setting of acute hemorrhage. The use of non-steroidal anti-inflammatory drugs was avoided.

Since laboratory results, including TEG, did not reflect the patient’s hemostasis defect, close intraoperative monitoring of bleeding status was performed by continually evaluating vital signs, examining blood loss in the operative field and suction canisters, and closely communicating with the obstetricians about uterine tone. The above-average blood loss was attributed to uterine atony due to observation by the surgeons, though incomplete hemostasis could not be ruled out completely. Postoperatively, close neurological exams for 24 hours to monitor for clinical signs of SEH were performed, including motor function test of both legs every four hours. Additionally, the patient had been informed about common symptoms of SEH, including rapid onset of pain, new onset of radiculopathy, and sensory and motor deficits of her lower extremities. In the event of such signs and symptoms, an emergent neuraxial MRI would be performed.

In summary, this case report highlights the importance of a multi-disciplinary approach. Close communication and coordination between the hematology, obstetric, and obstetric anesthesia services were crucial in achieving an uneventful CD in this patient with an otherwise extremely high bleeding risk.

## Data availability

All data underlying the results are available as part of the article and no additional source data are required.

## Consent

Written informed consent for publication of their clinical details and/or clinical images was obtained from the patient.
