# Supplementary material for: Case Report: Anesthetic management for Cesarean section in a parturient with unspecified inherited bleeding disorder
Source: F1000Res. 2018 Nov 29;7:1482. Originally published 2018 Sep 18. [Version 2] doi: 10.12688/f1000research.16097.2 (PMC6283378; doi:10.12688/f1000research.16097.2)
Supplement: Peer review history

# Peer review: Referee response for version 2

**DOI:** 10.5256/f1000research.18856.r41253

**Published:** 2018-12-05

**Authors:** Benjamin G. Cobb

**Affiliations:** 1 Department of Anesthesiology, University of North Carolina, Chapel Hill, NC, USA

The revised manuscript satisfactorily addresses all concerns described in my initial review.

I have read this submission. I believe that I have an appropriate level of expertise to confirm that it is of an acceptable scientific standard.

# Peer review: Referee response for version 2

**DOI:** 10.5256/f1000research.18856.r41252

**Published:** 2018-11-30

**Authors:** Allison J. Lee

**Affiliations:** 1 Department of Anesthesiology, Columbia University, New York City, NY, USA

The authors have satisfactorily addressed my concerns.

I have read this submission. I believe that I have an appropriate level of expertise to confirm that it is of an acceptable scientific standard.

# Peer review: Referee response for version 1

**DOI:** 10.5256/f1000research.17577.r38949

**Published:** 2018-10-17

**Authors:** Benjamin G. Cobb

**Affiliations:** 1 Department of Anesthesiology, University of North Carolina, Chapel Hill, NC, USA

The authors describe the use of regional anesthesia for cesarean section in a parturient with an unspecified inherited bleeding disorder. In this case, the clinical bleeding history in conjunction with the hematologic lab workup leading to the diagnosis of an ‘unspecified’ bleeding disorder is well described. The authors highlight both the importance of early multidisciplinary collaboration in the parturient with a bleeding disorder in an effort to minimize hemorrhagic complications. In addition, the role of informed consent for a patient with an unspecified bleeding disorder and unquantifiable risk of spinal/epidural hematoma should not be understated. Additional attention to the potential limitations of factor/platelet/fibrinogen replacement in the setting of *qualitative* coagulation defects to prevent hemorrhagic complications of neuraxial anesthesia may be warranted in the discussion. I commend the authors for describing the often challenging anesthetic risk-benefit assessment of a patient with an unspecified bleeding disorders.

  

- In addition to the cryoprecipitate transfusion, was von Willebrand factor/DDAVP administration considered given the unknown defect?
- Please clarify the institutional oxytocin protocol post-delivery. 
- Following the postpartum hemorrhage, what guided the decision to transfuse two additional units of platelets postoperatively and not other factors/fibrinogen?
- Is there any published literature on the prevalence of ‘unspecified bleeding disorders’ and/or standardization of these clinical diagnoses in the setting of normal coagulation workups by hematologists?
- Please clarify post-operative neurologic monitoring plan, including nursing neurological check frequency and patient surveillance counseling (concerning signs/symptoms).

I have read this submission. I believe that I have an appropriate level of expertise to confirm that it is of an acceptable scientific standard, however I have significant reservations, as outlined above.

# Response

**Published:** 2018-11-06

**Authors:** Laurent Bollag

**Affiliations:** University of Washington Medical Center, USA

Dear Dr. Cobb

Thank you for your comments to improve the quality of our manuscript.

I will reply point by point to your comments: 

- **In addition to the cryoprecipitate transfusion, was von Willebrand factor/DDAVP administration considered given the unknown defect?**

Willebrand factor/DDAVP administration was not given based on the norma; von Willebrand factor (VWF) parameters preoperatively.

 

- **Please clarify the institutional oxytocin protocol post-delivery. **

Our protocol is as follows: 15 IU per hour given on a drip. Once adequate uterine tone is achieved, the dose is reduced to 7.5  IU per hour, and again, once the tone is reported to be adequate, reduced  ca 50% (or 70ml /hr). This drip is continued postpartum.

 

- **Following the postpartum hemorrhage, what guided the decision to transfuse two additional units of platelets postoperatively and not other factors/fibrinogen?**

While uterine atony was the likely reason for the PPH, incomplete hemostasis we assumed and platelet substitution deemed the most appropriate therapeutic first step. 

- **Is there any published literature on the prevalence of ‘unspecified bleeding disorders’ and/or standardization of these clinical diagnoses in the setting of normal coagulation workups by hematologists?**

The lack of such literature prompted this report. 

 

- **Please clarify post-operative neurologic monitoring plan, including nursing neurological check frequency and patient surveillance counseling (concerning signs/symptoms).**

Local guidelines include vigilant neurological checks, including motor function of both legs every 4 hours for 24 hours. Patients are informed about the common symptoms of spinal nerve compression and the need to urgently report these to the nursing staff, namely:

1. rapid onset of pain
2. new onset radiculopathy
3. sensory and
4. motor function deficits

An emergent lumbar MRI will then confirm the diagnosis.

# Peer review: Referee response for version 1

**DOI:** 10.5256/f1000research.17577.r38948

**Published:** 2018-10-04

**Authors:** Allison J. Lee

**Affiliations:** 1 Department of Anesthesiology, Columbia University, New York City, NY, USA

This is a description of management of a challenging clinical scenario given the non-specific diagnosis of the bleeding disorder in this patient with a desire for neuraxial anesthesia.

 I was curious about the airway exam, considering that the team would have to prepare the possibility of inducing general anesthesia. I wonder if consideration was given to administering DDAVP or other treatment for von Willebrand's disease. Was an arterial line placed or considered, and what was the intravenous access in place?

 I am curious about the components of the intrathecal medications for the single shot spinal anesthetic. With the possibility for prolonged surgery, were any adjuvants, such as clonidine added to prolong the anesthetic?

 I would suggest editing the language in a few places. Instead of "epidurals", suggest instead, "labor epidural analgesia". Explain what is"routine oxytocin" in your institution. For what period postoperatively was the patient "closely monitored" and how? Were there nursing orders for frequent checks of neurologic status? Finally, I would suggest discussing the reasons for the low incidence of epidural hematoma in the obstetric population and including this reference: Lee L, et al ^1^.

I have read this submission. I believe that I have an appropriate level of expertise to confirm that it is of an acceptable scientific standard, however I have significant reservations, as outlined above.

## References

[1] LeeLOBatemanBTKheterpalSKlumpnerTTHouseyMAzizMFHandKWMacEachernMGoodierCGBernsteinJBauerMEMulticenter Perioperative Outcomes Group Investigators: Risk of Epidural Hematoma after Neuraxial Techniques in Thrombocytopenic Parturients: A Report from the Multicenter Perioperative Outcomes Group. *Anesthesiology*.126(6) : 10.1097/ALN.0000000000001630 1053-1063 10.1097/ALN.0000000000001630 28383323PMC5445642

# Response

**Published:** 2018-11-06

**Authors:** Laurent Bollag

**Affiliations:** University of Washington Medical Center, USA

Dear Dr Lee

Thank you for taking the time to review our case report to improve its quality!

I addressed your comments point by point below:

**I was curious about the airway exam, considering that the team would have to prepare the possibility of inducing general anesthesia. I wonder if consideration was given to administering DDAVP or other treatment for von Willebrand's disease. Was an arterial line placed or considered, and what was the intravenous access in place?**

Her airway exam was as follows: Mallampati Score 2, free head and neck movement (>6cm), normal mouth opening and no prominent incisors, in short pretty normal.

Willebrand factor/DDAVP administration was not recommended by the hematologist  based on the normal von Willebrand factor (VWF) parameters preoperatively.

She had a well working 18g IV. We decided not to place an Aline a priori, because we felt that it could be sited easily if need be. In cases where the placement is deemed technically complicated we do place A lines when a PPH is deemed likely, the same applies to a second large bore IV.

**I am curious about the components of the intrathecal medications for the single shot spinal anesthetic. With the possibility for prolonged surgery, were any adjuvants, such as clonidine added to prolong the anesthetic?**

We used out routine SPA mixture 12,5mg hyperbaric Bupivacaine, 10mcg Fentanyl and 100mcg PF Morphine. We did not add spinal clonidine, to avoid worsening of hypotension in a case where  a PPH was deemed likely. Clonidine could also be aded later IV, to extend spinal anesthesia duration, but again potentially worsen hypotension- for hours.

Obviously, we opted not to place a CSE in this potentially coagulopathic patient. 

**Explain what is"routine oxytocin" in your institution. For what period postoperatively was the patient "closely monitored" and how? Were there nursing orders for frequent checks of neurologic status? Finally, I would suggest discussing the reasons for the low incidence of epidural hematoma in the obstetric population and including this reference: Lee L, et al** **1**

Physiological hypercoagulability of pregnancy as well as the large epidural space in young parturients may explain why epidural hematomas in parturients are rare; additionally older guidelines are more conservative regarding platelet count cut-offs for neuraxial anesthesia.

The risk of performing neuraxial anesthesia in patients with platelet counts of less than 70,000 however remains unknown. **1**

We have addressed your other comments in our response to reviewer 1, who raised the same questions.

References

1. Lee LO, Bateman BT, Kheterpal S, Klumpner TT, Housey M, Aziz MF, Hand KW, MacEachern M, Goodier CG, Bernstein J, Bauer ME, Multicenter Perioperative Outcomes Group Investigators: Risk of Epidural Hematoma after Neuraxial Techniques in Thrombocytopenic Parturients: A Report from the Multicenter Perioperative Outcomes Group. *Anesthesiology*. 126 (6): 1053-1063 PubMed Abstract | Publisher Full Text
